# Integrating Pilot Study Findings into Larger Intervention Trials for Dementia Advance Care Planning

**DOI:** 10.1002/alz.095348

**Published:** 2025-01-09

**Authors:** Kara B Dassel, Jordana L Clayton, Eli Iacob, Rebecca Utz, Sara Bybee, Katherine P Supiano, Nancy R Aruscavage

**Affiliations:** ^1^ University of Utah, Salt Lake City, UT USA

## Abstract

**Background:**

Persons with dementia often lose decisional capacity before death, underscoring the need for dementia‐focused advance care planning tools. We created LEAD (Life‐Planning in Early Alzheimer’s and Other Dementias). This intervention guides patients with early‐stage dementia and their care partners through conversations to identify and discuss the patient’s values and preferences for end‐of‐life care. This intervention considers patients' preferences regarding when they have and do not have decisional capacity. It focuses on concordance between patient and care partner, facilitating care partners’ ability to be informed and confident proxy decision‐makers. This study aimed to evaluate the LEAD intervention’s feasibility and initial efficacy and discuss how this pilot work has informed the development and implementation of an NIH Stage‐I clinical trial.

**Method:**

Community‐based dyads (N = 51) completed the web‐based LEAD intervention and baseline, post‐intervention, three and six‐month post‐intervention surveys assessing decision‐making self‐efficacy (DMSE), relationship quality, and the completion of a medical advance directive. The study team analyzed participants' quantitative and qualitative feedback using the FRAME intervention modification model to identify areas of refinement for clinical trial implementation.

**Result:**

Patients and care partners were 61.8 (SD = 16.7) and 52.8 years (SD = 14.5) on average, respectively. Forty‐one percent of patients had a diagnosis of mild cognitive impairment, and 59% reported a family history of dementia. Thirty‐four dyads (66.7% retention) completed the nine‐month LEAD intervention. There was a significant increase in DMSE scores among patients and care partners (p<.05) and a decrease in relationship strain, as reported by care partners (p<.05). There were no changes in relationship quality. At baseline, 26 dyads (50.0%) had previously completed an advance directive; at six months post‐intervention, 12 dyads (23.5%) had completed this document. Participants provided feedback regarding the LEAD Guide wording, module instructions, conversation guidelines, and intervention length.

**Conclusion:**

Our findings suggest the LEAD Intervention is feasible and demonstrates initial efficacy in promoting dementia‐focused advance care planning. LEAD has the potential to help care partners improve their confidence as proxy decision‐makers when the patients can no longer advocate for themselves. Completing pilot work before more extensive clinical studies is essential in refining intervention usability, acceptability, and feasibility.

